# Characteristics and Causes of Particularly Major Road Traffic Accidents Involving Commercial Vehicles in China

**DOI:** 10.3390/ijerph18083878

**Published:** 2021-04-07

**Authors:** Mingwei Yan, Wentao Chen, Jianhao Wang, Mengmeng Zhang, Liang Zhao

**Affiliations:** 1 School of Transportation & Logistics Engineering, Shandong Jiaotong University, Jinan 250357, China; yanmingwei1116@163.com (M.Y.); zhangmengmeng@sdjtu.edu.cn (M.Z.); zhaoliang@sdjtu.edu.cn (L.Z.); 2 China Occupational Safety and Health Association, Beijing 100011, China; cwt123@vip.sina.com

**Keywords:** accident causation, individual, organization, road traffic, commercial vehicle

## Abstract

Major accidents occurred frequently in the road transportation industry, and the resulting harm to drivers, property loss, and traffic interruption are very serious. This study investigated 11 particularly major accidents involving commercial vehicles in China, and performed analysis on accident characteristics regarding the time, location, types of vehicles, and accident causation at different levels based on the 24Model. Large buses and dangerous goods vehicles were involved in 10 accidents and they all occurred on a freeway. The months from May to August, especially during the time periods of 2:00–4:00 and 14:00–16:00 every day, were the most prone to accidents. The driver’s speeding and fatigued driving, and vehicle failure were the direct causes of most of the accidents. The defects in organizational safety management involved 12 system elements, such as safety accountability, education and training, etc. Procedures are of no use if they were not followed, and there was often no effective process to assess the implementation of procedures in many organizations. The weaknesses in organizational safety culture were the source of accidents, which was mainly manifested in members’ inadequate cognition of key elements in the aspects of safety importance, safety commitment, safety management system, etc. Understanding the characteristics and root causes of accidents can help to prevent the recurrence of similar mistakes and strengthen preventative measures in road transportation enterprises.

## 1. Introduction

Road traffic accidents posing a serious hazard to modern societies, as they result in injuries, disabilities and even loss of lives, along with significant economic and social consequences. Globally, the number of deaths on the world’s roads remains unacceptably high, with around 1.35 million people dying each year [1]. Crash statistics showed that commercial vehicles present a more serious safety problem, particularly when considering the severity of losses in which they are involved [2]. The importance of commercial vehicles to passengers and freight logistics and the impact on the economic well-being of a country is well acknowledged; meanwhile, the impact that commercial vehicle crashes have on society is significant. In recent years, major or even particularly major accidents related to commercial vehicles have occurred frequently in China, e.g., a crash of a vehicle transporting hazardous goods that occurred on June 13 2020 killed 20 people and injured more than 170 [3].

In the context of road traffic safety, studying the factors that affect accidents and the associations between causal factors, especially those behind particularly major accidents, is very necessary. Many studies have applied safety analysis to explain the occurrence of road traffic accidents. The following stand out among the predictors of road traffic accidents due to their consistency in the literature: environmental factors such as the climate, weather and road infrastructure; mechanical factors such as the model, age and breakdowns of vehicles [4,5]; and individual driver factors such as the age [6,7], experience [8], driving style [9], safety belt use [10], alcohol consumption [11], fatigue [12,13,14] and chronic diseases such as obesity, anxiety, depression and personality disorders [15,16]. Recent evidence also has supported the existence of adverse task-related features in the occupational activity of professional drivers, such as irregular and prolonged work shifts [17,18], stressful work conditions [19,20] and problematic interactions with other road users [21,22].

Specifically, human factors and unsafe conditions are the primary causes of accidents. A previous study showed that human errors accounted for 95% of the causes of 5519 road traffic accidents in China from 2008 to 2012 [23]. Accidents caused by driver fatigue accounted for approximately 40% of major traffic accidents [24]. Other statistical data indicated that traffic rule violations were the main factors affecting road traffic safety [25]; thus, the control of these violations according to the laws and regulations can significantly reduce the occurrences of accidents. Additionally, external factors such as seasonal variations in weather can also influence road traffic safety; about 5% of road traffic accidents occurred because of factors related to various weather conditions [26]. This is in line with a variety of previous studies in which the road traffic accident rate depended on monthly and daily differences in weather conditions [27], such as the effects of rainfall, fog, windy weather and the amount of sunshine.

Despite previous studies on the frequency of road traffic accidents and its related causal factors, there is limited research about major or particularly major commercial vehicle accidents (i.e., transportation accidents) exploring the relationship between different types of causes. In China, commercial vehicles must be operated by an enterprise and are supervised by multiple external organizations. As a result, the causes of commercial vehicle accidents are more complex, and usually involve multiple organizations and deep causes at the organizational level [2]. Researchers have carried out preliminary analyses based on several major commercial vehicle crashes, and tried to identify unsafe behaviors at the individual level, management defects at the organizational level [24,25], and external driving environment factors [28,29]. However, people may not understand why the consequences were so severe and want desperately to find out what are the latent factors that caused the crash and led to the causalities.

What are the key points we should remember and how to avoid similar mistakes is of vital importance within the industry and for the whole world. Therefore, it is significant to create understanding about the nature of fatal traffic crashes, by identifying different salient factors that may influence the occurrence of accidents. The burden of most of these attributes can be reduced through effective interventions, which will result in a significant decrease in the burden of road transportation accidents. For that reason, this study aimed to provide a broad exploratory analysis for commercial vehicle accidents in China. Considering the typicality of the accident and the completeness of the investigation report, this paper mainly selected commercial vehicle accidents with relatively serious consequences for study.

Accidents can be explained differently, depending on various accident analytical methods. In the last few decades, several typical accident models, such as the Domino accident model [30], Swiss Cheese Model (SCM) [31], Human Factors Analysis Classification System (HFACS) [32], Systems-Theoretic Accident Model and Processes (STAMP) [33], etc., have dominated the safety research field literature, where they were widely used for accident analysis and prevention. Through comparison, it becomes evident that a common disadvantage of the present models lies in that they all fail to classify well the deep causal factors at the organizational level so that people may not prevent accidents by directly and accurately applying the analytical procedures and interpreting their results. Thus, the first objective of this study was to select an effective accident analysis approach based on the accident causation models and determine the causal factors at different levels. The second objective is to find out the common characteristics of fatal commercial vehicle accidents based on the typical indicators such as the time, location, season, etc. The last and most important objective is to identify the accidents’ dominant causes in the aspects of the individual factors and unsafe conditions, and further extricate the accidents’ latent causes regarding the organizational factors based on the analytical method.

## 2. Methodology

### 2.1. Cases Selection

Accident analysis/investigation is widely recognized as an important part of a comprehensive and efficient safety management [34]. Investigation reports provide multiple information about the accident: (a) the accident details such as the time, location, environment, people and vehicles involved, emergency rescue, etc.; (b) the causal factors such as some critical events, direct causes, indirect causes, etc.; (c) the involved organizations and corresponding penalty decisions; (d) the brief precautions for accident prevention. Indeed, the reports is generally too long to understand so it is necessary to extract the critical information for analysis and statistics. This study was performed based on the accident data obtained from the Ministry of Emergency Management of the People’s Republic of China (PRC, Beijing, China). A total of eleven particularly major accidents related to commercial vehicles that occurred within China during a period of ten consecutive years ranging from 2010 to 2019 were selected for analysis. The specific information regarding the selected accident cases can be seen in Table 1.

### 2.2. Analytical Approach

For every fatal accident, the basic information in the aspects of the time, location, vehicle, crash type, etc. was extracted from the investigation report, and then Excel software was used to carry out data statistics and presentation (e.g., time-series, location-series, etc.) for the trend or characteristics of the accident. In addition, graphic presentation techniques, such as fishbone diagrams and accident causation models were utilized in the analysis of the causes. Especially, the accident causation model played an important role in this work as it could demonstrate the logical relationships among different causal factors and help people better understand and remember the key lessons [35].

**Figure 1:**
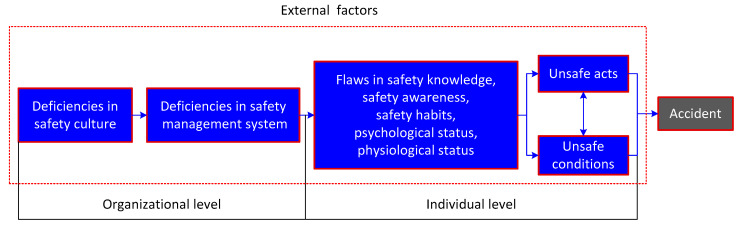
The accident causation model–24Model [34,36,37,38,39].

In this study, an improved causation model—24Model—which was established by our research team based on the domino accident theory and Swiss Cheese Mode (SCM) [36,37,38,39], was chosen as the tool for the analysis of commercial vehicle accidents, and its framework is shown in Figure 1. Here, “2” means causes at the individual level and organizational level; “4” means the causes classification, including unsafe acts and unsafe conditions, flaws in habitual behaviors, deficiencies in the safety management system, weaknesses in the safety culture. The 24Model indicates all accidents belonged to the organization type and were mainly attributed to internal organizational causes. The internal causes are much more controllable by the managers of the organization to achieve improvement in safety performance, so they usually serve as the key points for accident analysis. The external causes mainly involve factors from natural events, defective design, poor supervision from the government, deficiencies in laws or regulations, etc., which generally contribute to accidents by influencing the internal ones.

When utilizing the 24Model to analyze an accident, the first step is to find out the organization in which the accident occurred, and the following analysis begins from the bad outcomes (i.e., accident cases), to the identification of unsafe behaviors and unsafe conditions, to the mining of organizational defects regarding the safety management system and safety culture based on the individual factors, and finally to the external causes outside the organization.

The domino accident theory indicated that unsafe acts and unsafe conditions were the immediate causes of an accident [30,31]; furthermore, they were determined by various factors, such as individuals’ safety awareness, safety knowledge and safety habits [36,37], as well as their psychological and physiological status [38,39]. It is recognized that errors at the individual level are caused by root causes at the organizational level, i.e., the weaknesses in organizational safety management [40]. The safety management in an organization is implemented via a safety management system; therefore, the deficiencies in the safety management system, in turn, can be used as indicators to demonstrate the flaws in safety management. As shown in Table 2, fourteen key elements of management system for accident analysis have been summarized from the Occupational Health and Safety Management Systems (OHSMS), mainly involving safety policy, safety objectives, organizational structure and personnel allocation, safety accountabilities, training and education, resource management, emergency response planning, etc.

Safety culture, which reflects the belief, vision, value, or attitude shared by the staff related to safety, guides the establishment and implementation of the safety management system [41]; thus, bad safety culture or climate in an organization will lead to the deficient safety management system. Safety culture consists of many elements affecting the safety performance [40,41], mainly including the importance of safety, economic benefits of safety, demand of safety training, primary responsibility for safety, safety responsibility of managers, role of safety regulations, etc. Here, fourteen key elements have been summarized from the previous research and the specific descriptions are shown in Table 3.

## 3. Results and Discussion

This section carried out the process anatomy and cause identification for the 11 particularly major accident cases listed above. The common characteristics of these accidents in the aspects of vehicles, time, locations and crash types were analyzed and discussed, and various factors that led to accidents are identified, classified and displayed based on fishbone diagrams and 24Model.

### 3.1. Accident Characteristics

#### 3.1.1. Types of Vehicles and Crashes

Commercial vehicles are used to carry passengers and/or goods for profit. In China, commercial vehicles mainly include vehicles for ordinary goods, vehicles for dangerous goods, large buses, small taxis, etc. They must belong to an organization and are subject to safety supervision by the enterprise and the government. The particularly major accidents in this study involved three kinds of vehicles, including light goods vehicles, heavy semi-trailer tower vehicles and large buses, and two crashes also involved small private cars. Additionally, the crash forms of the vehicles include collision with central or roadside guardrail, rear-end collision and head-on collision, among of which, seven collisions further caused severe fires or explosions, and two vehicles rolled over and fell off the cliff.

**Figure 2:**
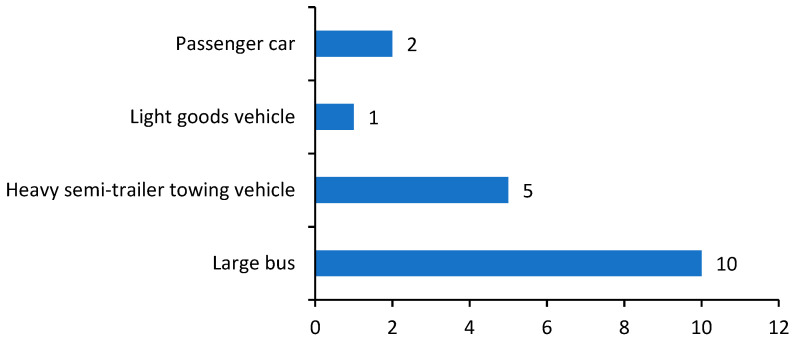
Number of different kinds of commercial vehicles involved in the crashes. Note: The vehicles in China are classified by the standard according to the purpose, wheel base, bodywork length, load, etc.

From Figure 2 we can see that large buses and dangerous goods vehicles (i.e., the light goods vehicle and heavy semi-trailer tower vehicle) appeared relatively frequently in the accidents. Especially, large buses were involved in almost all the accidents and often led to serious casualties due to their large passenger capacities, which is a main reason for the accident reaching a particularly major level. In addition, dangerous goods transportation vehicles appeared in five crashes. They are often loaded large quantities of dangerous chemicals such as the methanol, ethanol, etc., and when the vehicle crashed with other vehicles or road facilities, especially with the large buses, leakages occurred easily, and this triggered fires and explosions, or even a domino effect, thus causing more serious consequences.

#### 3.1.2. Time-Series Decomposition

**Figure 3:**
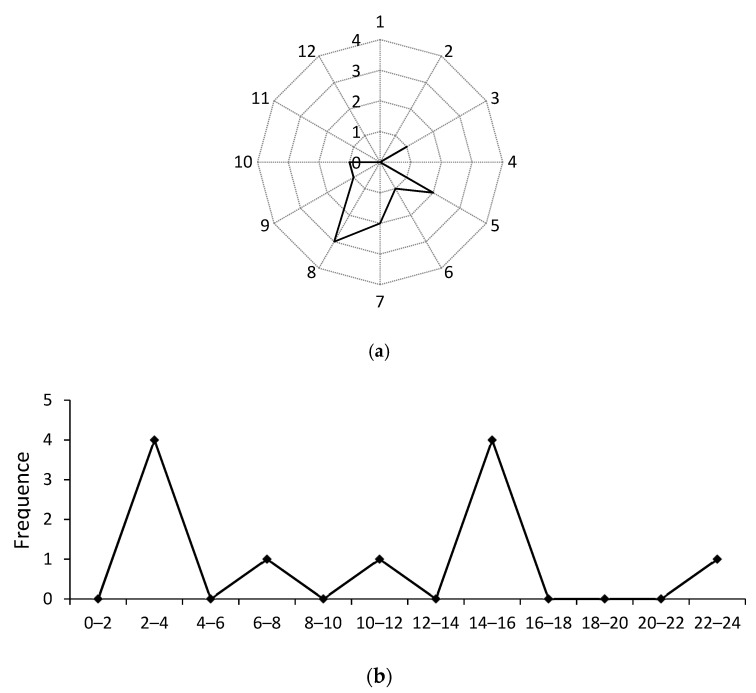
(**a**) Months. (**b**) Hours. Time-series analysis for commercial vehicle accidents.

The time series trend for particularly major accidents related to commercial vehicles clearly revealed the pattern of variable frequencies for different hours, months and seasons. In Figure 3a, it is observed that the months from May to August were most prone for driver accidents. During the months of June, July and August, the summer season starts with average temperatures warming up from 30 to 40 °C at the end of the season. The arrival of high temperature has great impacts on the driver’s physiological impact and pressure, and especially at 2:00–4:00, 14:00–16:00 every day, drivers are usually in a state of fatigue, so the two periods are the time of frequent accidents (see Figure 3b).

In addition, with the arrival of several holidays, more people travelling and the operation of bus business, the accident rate increased. Furthermore, schools were closed for summer season and many students and residents spent time indoors or leave the country for holiday. This increased the business of the commercial buses and led to significantly severe injuries and deaths during the summer season.

#### 3.1.3. Location-Series Decomposition

There were nine crashes that occurred on freeways. As we know, the traffic flow on the freeway is relatively large and the speed of vehicles is very fast, which is more dangerous than those ordinary low-speed urban and rural highways. Briefly, the freeway is the frequent area of major and particularly major road traffic accidents.

**Figure 4:**
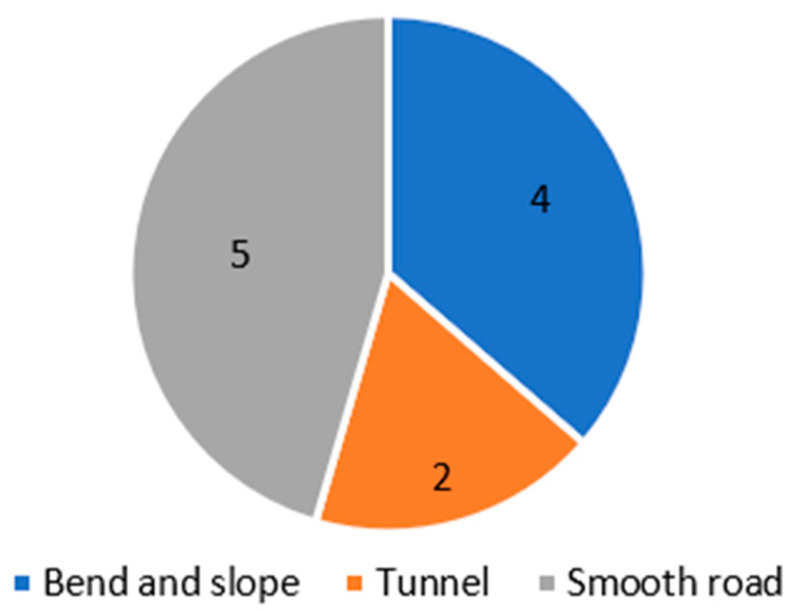
Location-series analysis for commercial vehicle accidents.

Four crashes occurred in areas of with steep bends and sloping roads on the freeway, and another two occurred at the entrance of a tunnel (see Figure 4). The linearity of both kinds of roads is relatively complex, which tended to affect the visual recognition effect of drivers or trigger the failure of the vehicles’ parts, thus causing the drivers’ wrong operation. Particularly, if the speed is too fast, the vehicle can easily lose control in an emergency, thus resulting in rear-ending, rollovers or other bad consequences. The road section of this series above is usually defined as an accident black spot, which is considered as the key area for the prevention of traffic accidents by government agencies.

### 3.2. Accident Causation

Generally, large-scale accidents occurred due to unexpected interactions among multiple failures. Based on the analysis of the accident characteristics, there was a nested collection of causal factors that triggered the crash. These causes ranged from straightforward ones in the aspects of drivers or vehicles to more fundamental roots such as managerial flaws or poor safety culture in the organization.

The road transportation system is composed of several important factors like the individual, vehicle, road, environment and organization. If there are defects in these sub-systems, accidental events will be easily triggered. Combined with the accident causation theory, the key causal factors of those particularly major traffic accidents related to commercial vehicles are extracted and classified in detail.

**Figure 5:**
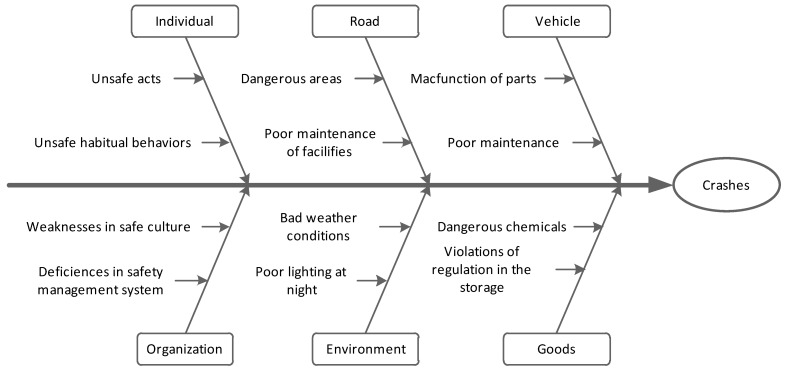
Fishbone diagram of the causal factors contributing to the commercial vehicle accidents.

In order to illustrate the role played by all causal factors, the fishbone diagram has been adopted for the accident causation analysis. The fishbone diagram is also called a cause and effect diagram and was often used to summarize the causes that create or contribute to a specific effect [42,43]. With a step-by-step in-depth study to analyze potential influencing factors, the fishbone diagram can systematize complicated incident causes. Accordingly, causal factors of those particularly major road transportation accidents have been categorized under six topics, as shown in Figure 5.

The further analysis and discussion regarding the sub-causes under the individual, road, vehicle, goods, environment, and organization categories will be carried out in the following section.

#### 3.2.1. Individual Factors

Through the investigation, all particularly major crashes in this study were classified as the “accountability accident”, namely human error accidents. This section has identified the individual’s unsafe acts leading to the accident and the flaws of the individual’s abilities in the aspects of the knowledge, awareness, habit, physiology and psychology that lead to the unsafe acts.

Ten categories of unsafe acts have been summarized from the accident report, as shown in Table 4. The speeding occurred most frequently, which was one of the most direct causes of crashes. In addition, the drivers’ fatigued driving and improper operation in an emergency were also the important causes of the accident.

Almost all unsafe acts were violations of regulations, involving different frontline workers like the drivers, passengers, and dynamic supervisors for vehicles. Additionally, unsafe decisions such as the inadequate safety training and poor safety inspection organized by the management had great impacts on frontline workers’ acts, which were usually considered as the potential causes of the accident. These causal factors will be discussed as the deficiencies at the organizational level in the following section.

**Figure 6:**
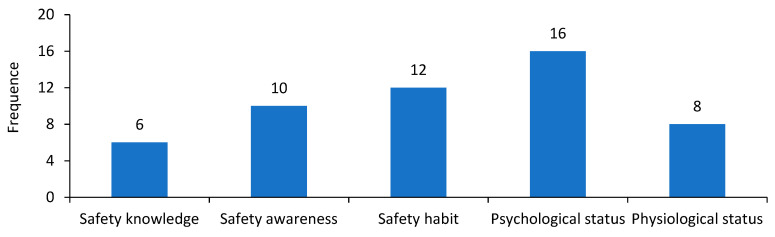
Statistics of unsafe habitual behaviors related to commercial vehicle accidents.

In Table 4, each unsafe act was caused by two or more flaws in drivers or managers’ habitual behaviors, and the specific statistics is shown in Figure 6. The organization should strengthen the management of individual in the aspects of training of knowledge and awareness, observation and correction of behaviors, regulation of physiological status, and intervention of psychological status.

- Inadequate safety knowledge: Drivers in several accidents lacked emergency driving experience, especially when facing bad road conditions on the freeway. In addition, some drivers and management did not know the characteristics of some hazardous chemicals loaded in the vehicle and did not know how to handle the leaks after the crash.
- Poor safety awareness: Some drivers were not aware of the serious consequences of speeding on the freeway and overloaded vehicles, and did not realize the risk of illegal transportation of hazardous chemicals. They failed to attach great importance on the implementation of rules or regulations regarding the traffic on the freeway.
- Poor safety habits: Some drivers had developed bad driving behaviors (e.g., speeding, fatigued driving, spinning the wheel sharply, reversing casually, etc.) in their daily work. Violations of regulations appeared very frequently in the driving as accidental events did not occur in the past and the organization did not remind or punish the drivers.
- Poor psychological status: Many drivers in accidents had unstable moods or bad psychology, such as the quick temper, low energy psychology (lazy, cutting corners), fluke mind, herd mentality, and some were almost not competent to do transportation work, as they were the main reason of unsafe acts in the driving.
- Poor physiological status: Fatigue is considered as one of the great taboos in the driving. Many drivers in accidents were very tired and distracted after the long-time driving and had a bad emergency capacity when facing a danger on the freeway.

#### 3.2.2. Unsafe Conditions

Unsafe conditions are also the important factors causing the commercial vehicle accidents. In the Fishbone diagram above, unsafe conditions regarding this kind of accident mainly involved the defects of the vehicle, goods, road, and environment. Vehicles and goods are usually managed and supervised by the transportation enterprises, and their unsafe conditions are determined as the internal factors according to the 24Model; while the management and maintenance of freeway and road facilities come from other organizations, and their unsafe states were usually classified as the external factors. The specific unsafe conditions leading to the commercial vehicle accidents are shown in Table 5.

Defects related to commercial vehicles appeared the most in these accident cases. The brake malfunction was very dangerous condition and it will easily lead to the severe crash once the vehicle encountered bad road conditions or environment. For vehicle overload (both goods and passengers), and defects of vehicle-mounted emergency facilities, both not only had indirect impact on the occurrence of the accident, but also led to the expansion of the accident scale and the casualties. Indeed, most of the unsafe conditions are directly or indirectly caused by unsafe acts; therefore, an effective way to eliminate unsafe conditions is to strengthen the control of the individual behaviors.

#### 3.2.3. Organizational Factors

The Chinese government has attached great importance to the safety supervision of commercial vehicles, especially for the large buses and dangerous goods vehicles. The daily operation of commercial vehicles is supervised jointly by multiple organizations. Commercial vehicles must rely on an enterprise to carry out transportation activities and the organization is responsible for the dispatching, inspection and maintenance of vehicles, as well as the online supervision of drivers. In addition, the transportation enterprises must apply for a legal production safety license and accept the supervision from different government agencies (e.g., emergency management, transportation management, traffic control, etc.). Thus, the occurrence of a commercial vehicle accident was often involved in factors from multiple organizations. The safety management defect within the organization is the root cause of the accident, while the safety supervision and improper decision-making outside the organization directly affect the safety work of the enterprise, which has important indirect impacts on the accident.

Considering the directness of the enterprise’s safety management to commercial vehicles and employees, this study focuses on the analysis of the shortcomings of safety work within the organization. Based on the 24Model, the deficiencies in the construction of organizational safety management system and safety culture elements have been identified from the accident report, which can reflect the weaknesses in the safety supervision of related government agencies.

- Deficiencies in the safety management system

Organizational safety management determines individual behaviors [36,37]. According to the statistical analysis regarding unsafe behaviors and unsafe conditions, the defects in safety management of transportation enterprises are identified or inferred, mainly involving a total of 12 system elements (see Table 6). Common problems in organizational safety management system mainly involved the elements such as the organizational structure and personnel allocation, safety commitment or responsibility, safety education and training, identification of hidden dangers and risk assessment, etc. Procedures are of no use if they were not followed, and there was often no effective process to assess the implementation of procedures, regulations and rules in many accident organizations.

Indeed, the deficiencies in the organization’s safety policy and safety objective were not easy to be identified from the accident report, but it can be inferred that the safety policy of those accident enterprises failed to properly reflect the importance attached to safety. Although the organization may set its own safety goal, it failed to formulate effective measures to accomplish the goal. As a result, the risk identification and rectification rate, safety education and training rate, the number of emergency drills and other aspects did not meet the requirements of relevant laws and regulations.

- Deficiencies in the safety culture

Safety culture is considered as the guiding ideology (i.e., value, belief, vision, etc.) for the enterprise’s safety management. The weaknesses in organizational safety culture were the source of accidents, which is mainly manifested in members’ inadequate understanding and cognition for safety culture elements, thus failing to form good safety climate in the organization. Based on the above analysis of accident causation at different levels, the weaknesses in the construction of safety culture in accident organizations are identified, and five categories regarding the elements are summarized as follows:

(1) Recognition for safety importance

The management and drivers did not attach great importance to safety, did not put safety work in the first place, did not integrate safety work into the normal management of the enterprise, and believed that safety was only input and could not produce economic benefits. Members in these business units did not reach a consensus on safety beliefs such as “safety is the first priority”, “economic benefit of safety”, “integration of safety and management”, “treatments for safety performance”.

(2) Recognition for safety commitment

The top managers, middle management, line management and front-line operators of the enterprise failed to realize and understand their own post responsibilities, and did not well fulfill their safety responsibilities and commitments signed level by level. Members in these business units did not reach a consensus on safety beliefs such as “primary responsibility for safety”, “safety responsibility of the management”, “role of the safety department”, “responsibilities of line management departments”.

(3) Recognition for safety management system

The organization did not realize the important influence of safety management system on individual unsafe behaviors. The members did not write the safety work into procedures in accordance with the requirement of laws and regulations, and the implementation of the systems were not completely recorded, which failed to realize the whole process management of enterprise safety including pre-prevention, in-process emergency response and post-investigation and analysis. Members in these business units did not reach a consensus on “role of safety laws and regulations”, “role of safety management system”, “emergency capability”.

(4) Recognition for safety practice

It is meaningless if safety rules and regulations only remain on paper. The organization shall ensure the safety investment of human resources and capitals in production according to the specific requirement of laws and regulations, formulate effective execution measures regarding the procedure documents, and especially, strengthen the assessment and feedback of the implementation effect. Members in these business units did not reach a consensus on “demand of safety education and training”, “satisfaction for facilities and workplace”, “safety performance and human resources”, “types of safety inspections”.

## 4. Conclusions

This study performed an analysis on 11 particularly major commercial vehicle accidents, focusing more on the identification and presentation of common characteristics and causes at different levels. The 24Model which was improved from different accident models was used in the accident analysis, and especially, the causal factors at the organizational level regarding the safety management system and safety culture were newly summarized for the identification of latent causes. Thus, several important findings were summarized and they can provide a basis for the development of accident prevention measures for the government and enterprise.

The basic accident information in the aspects of the time, location, vehicle, crash type, etc. was extracted from the investigation report, and the trends or characteristics were summarized and discussed. Large buses and dangerous goods vehicles were involved in 10 accidents and should be supervised strictly by the government. The months from May to August, especially at the time of 2:00–4:00, 14:00–16:00 every day, were most prone for driver accidents. Freeways is the frequent location of particularly major road traffic accidents.

Unsafe acts and unsafe conditions were the immediate causes of the accidents, moreover, almost all unsafe acts were violations of existing driving regulations. Speeding occurred most frequently, which was one of the most direct causes of the accidents. In addition, drivers’ fatigued driving and improper operation in an emergency had great impact on the occurrence of the crashes. Unsafe conditions related to commercial vehicle maintenance such as malfunction of the brake system and overloading of vehicles appeared the most of the accident cases.

More efforts should be taken on the root causes at the organizational level. Common problems in safety management system mainly involved the elements such as the organizational structure and personnel allocation, safety commitment or responsibility, safety training, etc. Procedures are of no use if they were not followed, and there was often no effective process to assess the implementation of safety procedures in many accident organizations. The weaknesses in organizational safety culture were the source of accidents, which was mainly manifested in members’ inadequate cognition for elements in the aspects of safety importance, safety commitment, safety management system and safety practice.

## 5. Limitations and Future Works

The object of this study is particularly major commercial vehicle accidents, so there have been few cases in recent years, and the laws of some research results, especially the statistics of accident characteristics, are not obvious. Therefore, it is necessary to carry out further research using a larger sample of general commercial vehicle accidents, and to make a comparative analysis with the present results. In addition, this study focused more on the characteristics and causes of the accidents, while the corresponding accident prevention measures in the aspects of the government, enterprises and individuals were less involved, which will also be the focus of the next research.

## Figures and Tables

**Table 1:** The particularly major accident cases related to commercial vehicles within China.

No.	Year	Location	Description	Deaths	Injuries
1	2019	Changchun–Shenzhen Freeway	Collisions between a coach, crash barriers, and an opposite truck	36	36
2	2017	Beijing–Kunming Freeway	Collision between a coach and the headwall of a tunnel portal	36	13
3	2016	Yizhang–Fengtouling Freeway	Collision between a coach and crash barriers, and further causing a fire	35	13
4	2015	County Highway	Fall of a coach off a cliff	35	11
5	2014	Shanghai–Kunming Freeway	Collision between a coach and a truck loaded with ethanol, and further causing a fire	58	2
6	2014	National Highway	Collision between a coach and an off-road vehicle, and further falling off a cliff	44	11
7	2014	Jincheng–Jiyuan Freeway	Collision between two articulated vehicles loaded with methanol, further causing a deflagration	40	12
8	2012	Baotou–Maoming Freeway	Collision between a coach and a truck loaded with methanol, further causing a deflagration	36	3
9	2011	Binhai–Baoding Freeway	Collision between a coach and a car	35	19
10	2011	Beijing–Zhuhai Freeway	Deflagration of a coach loaded with dangerous goods	41	6
11	2010	Changchun–Shenzhen Freeway	Collision between a coach and a truck, and further causing a fire	33	24

Note: In China, an accident which kills more than 30 people is classified as a particularly major accident by the government.

**Table 2:** Key elements in the development of safety management system (SMS).

No.	Elements	Descriptions
1	Safety policy	The safety policy is a set of principles stated as commitments in which top management outlines the long-term direction of the organization to support and continually improve its safety performance. It provides an overall framework for the organization to set objectives and take actions to achieve the intended outcomes of the SMS.
2	Safety objective	Objectives are established to maintain and improve safety performance. The safety objective of an enterprise should be stricter than the requirements in the government’s regulations. The contents should be refined and quantified as much as possible, e.g., emphasizing the prevention of major accidents, setting acceptable accident rates, etc.
3	Organizational structure and personnel allocation	An organization should establish a health, safety & environment (HSE) department and allocate enough technical personnel according to the scope, size, and complexity of operations. For example, the Work Safety Law of the PRC stipulates that a transportation enterprise must set a special HSE department and allocate full-time safety engineers.
4	Safety accountability	Members in the organization should understand their role and authorities for achieving the intended outcomes of the SMS. The safety manager has the responsibility for ensuring the effective implementation of SMS; every person in the workplace needs to take account not only of their own safety, but also the safety of others.
5	Safety regulations and systems	An organization must establish operational procedures that are appropriate to the actual working situation and in accordance with safety policies. These procedures should be documented and constantly updated. The safety practices of operators can be conveyed to the entire organization through these documentations.
6	Hazard identification and assessment	An organization should establish a closed-loop program to ensure the identification of hazards, assessment of risks, development and implementation of measures. Risk controls can be any changes to the system, including adding or changing procedures, improving training contents, increasing or modifying facilities, etc.
7	Education and training	An organization should develop and maintain a safety training program that imbues the personnel with safety knowledge and capabilities. The main types of training include induction training and regular training: induction training helps personnel understand their safety responsibilities, while regular training is used to maintain their job skills.
8	Resources management	An organization should develop and maintain a resource management system that provides human resources (e.g., personnel selection and arrangement) and enough finance (for safety operations, PPE purchase, facilities maintenance, etc.). In resource management, safety should take priority over other concerns.
9	Safety management information system	An organization should collect information to guarantee its safe operation, which can be get from many sources, including the monitoring of daily activities, incidents investigation, employee reports, and feedback systems. For example, the commercial vehicles must be equipped with a dynamic monitoring device by the enterprise.
10	Interested parties	An organization should be responsible for the safety management of the personnel from other interested parties. Interested parties include: (a) parent organizations, (b) suppliers, contractors and subcontractors, (c) employers’ organizations, (d) owners, shareholders, visitors, local community and the general public, etc.
11	Safety communication	An organization should provide communication channels that allow employees to communicate about the organizational safety objectives, special safety actions, and safety-critical information with managers or other employees. The communication processes should provide for the gathering, updating and dissemination of information.
12	Emergency response plan	An organization should develop effective emergency response plans and they must be in writing to identify the responsibilities of the management, principal operators, and other employees. Emergency exercise should be held regularly to verify the practical effectiveness of the plan and ensure its continuous improvement.
13	Accident report and investigation	An organization should establish a procedure for accident report and investigation. The accident investigation results should be published to the society timely and useful lessons should be summarized to be as important contents in the education and training for members in the organization.
14	Continuous improvement	An organization should develop and maintain a comprehensive safety performance monitoring system. This system requires regular implementation to assess safety performance compliance with the safety objectives. This helps to eliminate the causes for poor performance and continuously improve the safety objectives.

**Table 3:** Key elements in the construction of safety culture.

No.	Elements	Descriptions
1	Importance of safety	This means safety is the first priority in the practical operation. The Chinese safety production policy is “safety first and precaution crucial.” This reflects the attitude of the staff toward the relationship between safety, production, efficiency, and profit.
2	Economic benefit of safety	When the managers recognize investing in safety can bring economic benefits, they will voluntarily invest in distributing safety resources. The economic benefits of safety works are mainly manifested as decreased casualties and losses from accidents.
3	Integration of safety and management	Safety is a priority consideration in each work activity for a project. The Work Safety Law of the PRC stipulates, “facilities related to safety and health must be designed, constructed, and put into operation corresponding with the main project during its construction, renovation, and expansion”.
4	Primary responsibility for safety	Safety is the responsibility of both the organizations and individuals. If the personnel do not regard safety as their own responsibility, the safety performance cannot be maintained or enhanced.
5	Safety responsibility of managers	Managers set examples for the staff. They control organizational resources and affect safety performance through their behaviors and leadership. Therefore, when managers take charge of greater responsibilities for safety issues, the organization will achieve better safety performance.
6	Role of safety laws and regulations	Safety laws and regulations are derived from multiple accidents and must be enforced without any compromise. They are necessary but insufficient conditions for achieving safety objectives. The real operating conditions must exceed the requirements in the laws and regulations.
7	Role of safety management system	The organization should recognize that SMS can help minimize occupational risks, prevent accidental events, and handle emergency situations. Safety management works must be consistent with the policies, objectives, procedures, and recordable information that are designed in system documents.
8	Role of the safety department	The safety department plays the role of organization, coordination, consultation and supervision in the safety management of an organization. It should not be first blamed when accidents occurred. However, the safety department must have professional personnel and can provide high-quality advice conducive to organizational safety.
9	Responsibilities of line management departments	Departments within an organization assume the primary responsibility for their own safety performance (rather than the special safety department). If the departments take more proactive activities, better safety performance will be achieved for themselves.
10	Demand for safety education and training	Safety training is a mandatory requirement in the Chinese law. The degree of safety training demand reflects the effectiveness of the previous safety training. The more effective the safety training, the more the demand for a subsequent training. Generally, the degree of demand for safety training reflects individuals’ safety awareness and knowledge.
11	Satisfaction for facilities and workplace	The less satisfied the employees are with the safety and reliability of the facility and workplace, the higher the safety awareness they have. This means if the employees have a higher safety expectation and demand, the organization will achieve better safety performance.
12	Safety performance and human resources	Good human resources will help the organization to improve the safety performance. New employees need to be selected based on their safety knowledge, capabilities, and skills. For the promotion of employees, their safety performances should be taken into consideration.
13	Types of safety inspections	The organization shall perform systematic safety inspections for the “hardware” and “software” in the workplace. The main types of safety inspections include the routine inspections, regular or unscheduled inspections, comprehensive inspections, and special inspections, etc.
14	Emergency capability	The emergency capability for individuals is the ability to quickly recover the organization from an emergency to safe situation. It is manifested in the emergency plans that determine everyone’s responsibilities in an emergency status. The emergency plan requires regular exercises.

**Table 4:** Individual factors related to the commercial vehicle accidents.

No.	Unsafe Act	Frequency	Corresponding Flaws in Habitual Behaviors	Violations
1	Speeding	6	Safety awareness Safety habit Quick temper	Article 42 in the Road Traffic Safety Law of the People’s Republic of China (PRC): A motor vehicle driving on a road shall not exceed the maximum speed indicated by the speed limit sign.
2	Fatigue driving	4	Safety habit Fatigue	Article 25 in the Measures of Dynamic Supervision for Road Transport Vehicles: The commercial vehicle drivers must rest for at least 20 min after a two-hour driving on the night shift.
3	Improper operations in an emergency (e.g., spinning the wheel sharply, not braking timely)	4	Safety knowledge Fatigue Distraction	These were a series of high-risk acts of the drivers that violated the organizational standard operating procedure that stipulates the matters needing attention in the operation of the driving.
4	Illegal loading and transportation of dangerous chemicals (without license)	2	Safety knowledge Safety awareness Safety habit Fluke mind	Article 23 in the Regulations on Road Transport of Dangerous Goods: It is prohibited to use vehicles that are modified without permission, that fail to meet the technical level stipulated by the government for the transport of dangerous goods.
5	Failure to follow the right lane	2	Safety habit Safety awareness	Article 44 and 48 in the Regulations for the Implementation of the Road Traffic Safety Law of the PRC: A vehicle shall slow down and keep to the right side when encountering a vehicle in a relative direction; A vehicle that changes lanes shall not affect other vehicles in the adjacent lanes.
6	Failure to use a seat belt (passengers)	2	Safety awareness Herd mentality	Article 51 in the Road Traffic Safety Law of the PRC: When a motor vehicle is moving, the driver and passenger shall use the seat belts.
7	Reversing on the freeway	1	Least energy psychology	Article 82 in the Regulations for the Implementation of the Road Traffic Safety Law of the PRC: A motor vehicle driving on a freeway shall not reverse, go the wrong side, make a u-turn across the central divider or stop in the lane.
8	Driving slowly on the freeway (ramp)	1	Safety awareness Safety habit	Article 78 in the Regulations for the Implementation of the Road Traffic Safety Law of the PRC: The maximum speed on a freeway shall not exceed 120 km/h, and the minimum speed shall not be less than 60 km/h.
9	Failure to check the vehicles before driving	1	Safety habit Least energy psychology	Article 21 in the Road Traffic Safety Law of the PRC: The driver shall seriously inspect the safety and technical performance of the vehicle before driving; it is prohibited to drive vehicles with hidden risks, such as those with incomplete safety facilities or whose parts do not meet technical standards.
10	Failure to report the violations timely (speeding and fatigue driving)	4	Safety habit Safety awareness	Article 26 in the Measures of Dynamic Supervision for Road Transport Vehicles: The monitoring personnel shall remind the driver to correct violations timely; if the driver continued driving illegally, the monitoring personnel should report to the organization and the line management shall take immediate measures to stop it.

**Table 5:** Unsafe conditions leading to the commercial vehicle accidents.

No.	Category	Frequency	Subcategory
1	Vehicles	18	Overloading of goods vehicles or large buses (8); Malfunction of brake system (3); Illegal modification of vehicles (increasing seats in the bus or increasing a cargo compartment in the goods vehicle) (2); Malfunction of dynamic monitoring system (2); A lack of the emergency shutoff device in the tank truck (1); Missing of emergency hammers (1); Tire burst (1).
2	Goods	3	Illegal storage and transportation of hazardous chemicals in goods vehicles or large buses (2); Rough packaging of dangerous goods (1).
3	Road	3	Accident blackspot (2); Abrasion of the boundary between two lanes (1).
4	Environment	1	Dark environment around the accident site (not turning on the street lights).

**Table 6:** Deficiencies in the safety management system leading to the commercial vehicle accidents.

No.	Elements	Frequency	Description
1	Safety policy	N/A	The deficiency in safety policy is hard to identify from the report, while we can induce the safety policy in those accident organizations must not put safety in the first place in production, and did not reflect the importance of safety and the safety commitments of the top management.
2	Safety objective	N/A	Some organizations have set their own objectives in the aspects of accident rate, violations, safety training, hazard rectification, etc. However, the objectives were not well decomposed into different departments and the personnel were not aware of their role in the realization of objectives.
3	Organizational structure and personnel allocation	7	Some organizations did not set up a special safety management department or did not allocated full-time persons for the work safety. The number of dynamic monitoring staff did not meet the requirement and the professional certification of some employees such as the drivers, escorts of goods vehicles and vehicle detection staff was expired.
4	Safety accountability	9	The top management did not assume the primary responsibility for safety and failed to put safety in the first place. The driver was the direct responsible person of the accident due to their violations of regulations in the driving. The safety managers did not enhance the supervision and training for the driver and escort, and did not find the defects of the vehicles timely. The employees in charge of the dynamic monitor for vehicles did not remind the drivers or report to the management regarding the violations and some even took off the post without authorization.
5	Safety regulations and systems	11	Some organizations violated laws or regulations in the refit of the vehicles and the transportation of hazardous chemicals (no license). They developed many procedures for safety management but some were not practical and there was no process to assess the implementation effect, e.g., the work and rest system, dynamic monitoring system for vehicles.
6	Hazard identification and assessment	7	The drivers or maintenance men did not carry out routine hazard identification for vehicles and failed to find the defects or risks in the brake system, tyres, accessory safety device, etc. The top management did not urge people to realize the close loop of identification, analysis, assessment and elimination for the hazards.
7	Education and training	8	Some organizations did not carry out the pre-job safety training for new employees or the time of the regular safety training for old employees was insufficient.
8	Resources management	3	Some organizations did not attach importance to the information system in safety management. There are three vehicles that were not equipped with the dynamic monitoring system according to the requirement of the Chinese law.
9	Interested parties	5	Some transportation enterprises often rented vehicles to individuals or groups, but they did not perform a strict safety supervision for those parties in the safety education of drivers, hazard identification of vehicles, dynamic monitoring of employees and vehicles.
10	Safety communication	4	There is no effective channel for the drivers to communicate with the management regarding the time of work and rest.
11	Emergency response plan	3	Some organizations had their own emergency response plan but the exercise regarding the emergency in different situation was not carried out as expected.
12	Accident report and investigation	2	Similar accidents had occurred in two organizations before the crashes listed in Table 1. However, the organizations failed to carry out an effective accident investigation and analysis, and useful lessons learned from the accident were not well notified to the employees.

## Data Availability

The data presented in this study are available on request from the corresponding author.
